# Entropy Analysis of Heart Rate Variability in Different Sleep Stages

**DOI:** 10.3390/e24030379

**Published:** 2022-03-08

**Authors:** Chang Yan, Peng Li, Meicheng Yang, Yang Li, Jianqing Li, Hongxing Zhang, Chengyu Liu

**Affiliations:** 1 School of Instrument Science and Engineering, Southeast University, Nanjing 210096, China; meicheng@seu.edu.cn (M.Y.); liy100@foxmail.com (Y.L.); ljq@seu.edu.cn (J.L.); 2 Division of Sleep and Circadian Disorders, Brigham and Women’s Hospital, Harvard Medical School, Boston, MA 02115, USA; pli9@bwh.harvard.edu; 3 State Key Laboratory of Proteomics, Beijing Proteome Research Center, National Center for Protein Sciences, Beijing Institute of Lifeomics, Beijing 102206, China; zhanghx08@126.com

**Keywords:** entropy, heart rate variability (HRV), complexity, sleep stage

## Abstract

How the complexity or irregularity of heart rate variability (HRV) changes across different sleep stages and the importance of these features in sleep staging are not fully understood. This study aimed to investigate the complexity or irregularity of the RR interval time series in different sleep stages and explore their values in sleep staging. We performed approximate entropy (ApEn), sample entropy (SampEn), fuzzy entropy (FuzzyEn), distribution entropy (DistEn), conditional entropy (CE), and permutation entropy (PermEn) analyses on RR interval time series extracted from epochs that were constructed based on two methods: (1) 270-s epoch length and (2) 300-s epoch length. To test whether adding the entropy measures can improve the accuracy of sleep staging using linear HRV indices, XGBoost was used to examine the abilities to differentiate among: (i) 5 classes [Wake (W), non-rapid-eye-movement (NREM), which can be divide into 3 sub-stages: stage N1, stage N2, and stage N3, and rapid-eye-movement (REM)]; (ii) 4 classes [W, light sleep (combined N1 and N2), deep sleep (N3), and REM]; and (iii) 3 classes: (W, NREM, and REM). SampEn, FuzzyEn, and CE significantly increased from W to N3 and decreased in REM. DistEn increased from W to N1, decreased in N2, and further decreased in N3; it increased in REM. The average accuracy of the three tasks using linear and entropy features were 42.1%, 59.1%, and 60.8%, respectively, based on 270-s epoch length; all were significantly lower than the performance based on 300-s epoch length (i.e., 54.3%, 63.1%, and 67.5%, respectively). Adding entropy measures to the XGBoost model of linear parameters did not significantly improve the classification performance. However, entropy measures, especially PermEn, DistEn, and FuzzyEn, demonstrated greater importance than most of the linear parameters in the XGBoost model.300-s270-s.

## 1. Introduction

The autonomic nervous system (ANS) plays an important role in regulating sleep cycles [1,2,3,4]. There are mainly two types of sleep: non-rapid eye movement (NREM) sleep and rapid eye movement (REM) sleep [5]. NREM can be further divided into three stages, i.e., stage 1, 2, and 3, which are usually named N1, N2, and N3, respectively. The four sleep stages (i.e., N1, N2, N3, and REM) are cycled through multiple times smoothly within a regular night of sleep. The parasympathetic (vagus) nerve has been found to be important in organizing sleep and wakefulness [6]. During NREM sleep, parasympathetic activity predominates, and the heart rate decreases. In contrast, sympathetic nerve activity results in heart rate acceleration in REM sleep [1]. Therefore, understanding the changes of the ANS in different stages of sleep is essential for accurately assessing an individual’s sleep quality.

Heart rate variability (HRV), the tiny variation of RR intervals from an electrocardiogram (ECG), has been widely applied as a non-invasive method to evaluate ANS modulation [7,8]. Danguolė et al. demonstrated that the heart rate decreased during sleep stages N1, N2, and N3 and increased in REM sleep [9]. By using spectral components of HRV, Toscani et al. showed that the power of the high-frequency (HF) component of HRV significantly increased in NREM [10] and was particularly elevated in N3 [11]. The ratio of low-frequency and HF (LF/HF) reduced in NREM [12,13]. In addition, the total power of the HRV spectrum and the power of the very low frequency component were both significantly higher in REM sleep [14].

Physiological systems are complex, nonlinear, and nonstationary partially due to the mutual coupling between multiple subsystems [15,16]. The cardiovascular control system also has complex dynamical behavior and nonlinear characteristics. The nonlinear and nonstationary information hidden in the ECG signals and RR interval time series cannot be obtained by traditional linear methods [17]. Nonlinear measures may provide valuable insights in addition to the time- and frequency-domain features by evaluating the complexity or irregularity of time series [18]. Among others, entropy indices are commonly used, such as approximate entropy (ApEn) [19], sample entropy (SampEn) [20], fuzzy entropy (FuzzyEn) [21], conditional entropy (CE) [22], distribution entropy (DistEn) [18], and permutation entropy (PermEn) [23]. In prior studies, the SampEn of HRV in the REM stage was shown to be similar to that during wakefulness, whereas it was increased during deep sleep [24]. Based on a multiscale analysis framework using entropy, it was shown that the complexity of HRV during wakefulness and REM was lower than that in NREM [25]. In another study, ApEn decreased from W to NREM and decreased further in REM, whereas SampEn decreased from W to N2, then increased at N3 and decreased again in REM [26].

In recent years, HRV measures have also been combined with computer learning models for sleep staging [27,28,29,30,31,32]. In these studies, mainly linear HRV features were used as inputs. Moreover, the classification was usually based on a 300-s epoch, and only the 300-s windows that covered a single sleep stage were used; the 300-s windows that involved more than one sleep stage were excluded [31]. In other words, many epochs would be removed because they failed to meet the criterion, and the chosen epochs were discontinuous.

The complexity of HRV in different sleep stages is not fully understood. It is yet to be determined how HRV complexity changes with 30-s as the step size, an approach that is recommended in sleep staging by the American Academy of Sleep Medicine (AASM). In the current study, we sought to: (1) study the complexity of HRV in different sleep stages by entropy algorithms; (2) investigate whether adding entropy features to models including only linear measures can improve the accuracy of sleep staging; and (3) compare the performance of two difference analysis schemes using respectively a 300-s epoch length and a 270-s epoch length270-s300-s. Based on a prior study [24], the RR interval time series at NREM has the highest SampEn (i.e., highest irregularity or lowest complexity). We thus hypothesized that the complexity of heartbeats during NREM sleep is lower than that during W and REM. We further hypothesized that during N3 sleep, the complexity of RR interval time series would be the lowest (i.e., the irregularity would be the highest). In addition, we also expected to observe better performance in general using the 300-s epoch approach since the structure of the 300-s epoch should be relatively consistent.

## 2. Materials and Methods

### 2.1. Materials

The MIT-BIH Polysomnographic Database was used, which is publicly available from the PhysioBank Archives [33,34]. It consists of 18 recordings of multiple physiologic signals during sleep collected from 16 male subjects (age: 32–56, weight: 89–152 kg). The database contains over 80 h of polysomnographic recordings with sleep stages scored already. All recordings were digitized at a sampling frequency of 250 Hz and the ECG signals were annotated beat-by-beat. Each recording comes from a separate participant except that the files “slp01a” and “slp01b”, and “slp02a” and “slp02b” were collected from the same person. “slp01a” and “slp01b” were merged, as were “slp02a” and “slp02b”. Therefore, the data used in this paper was 16 independent recordings.

The RR intervals to be analyzed were constructed with the annotated R waves in this database. The time series was constructed as follows.

**Figure 1:**
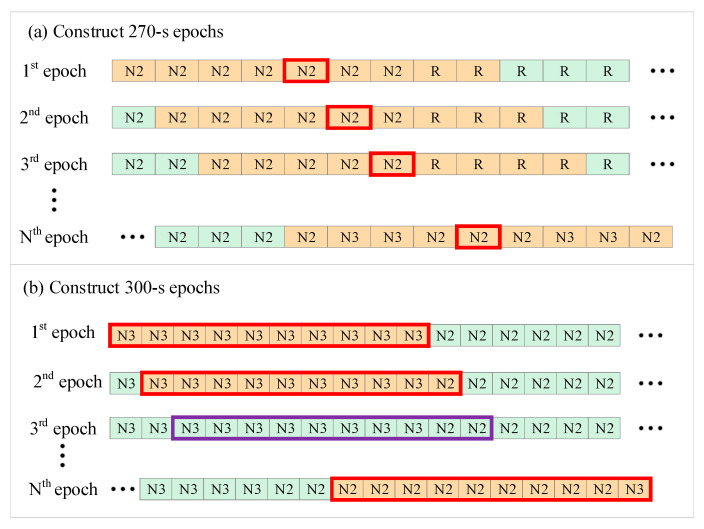
The schematic of 270-s and 300-s epoch construction. Each small square represents a 30-s epoch, and the content in the square represents the sleep stage annotation. The sub-epochs painted in orange constitute the currently constructed epoch. (**a**) A window of 270-s in length slides forward with 30-s as the step length. The red box represents the middle 30-s sub-epoch, which represents the annotation of the current 270-s epoch. (**b**) A window of 300-s in length slides forward with 30-s as the step length. The red boxes indicate the current 300-s epoch was reserved, while purple boxes indicate that it has been excluded. The RR interval series corresponding to the current 270-s or 300-s epoch is the time series to be analyzed.

- For 270-s epoch length:

Nine consecutive sub-epochs, each of 30-s in length, were concatenated to make one epoch of 270-s. The sleep stage of the 270-s epoch was set to the same as the middle 30-s sub-epoch270-s. The window slid forward in a 30-s time step to construct the next 270-s epoch with an overlap of 240-s with the preceding window. The method is summarized in Figure 1a.

- For 300-s epoch length:

A 300-s sliding window that moved forward in steps of 30-s with an overlap of 270-s between consecutive windows was used. The sleep stage of the current epoch was defined based on which sleep stage dominated the ten 30-s sub-epochs300-s, i.e., if more than 8 of the ten 30-s sub-epochs belonged to the same stage, the 300-s epoch was labeled with this specific stage. Otherwise, the 300-s epoch was excluded from the analysis. The method is summarized in Figure 1b.

Every RR interval with a length of 270-s or 300-s was preprocessed to eliminate the effect of anomalous intervals on entropy analysis based on the algorithm used in a previous study [34]. Firstly, if an RR interval was greater than the median value of five surrounding RR intervals, it would be identified as a spike. The spikes were corrected by an impulse rejection filter [35]. Secondly, any RR intervals that were greater or less than the global average ± three standard deviations (moving standard deviations with window length 100 RR intervals) were removed [36].

### 2.2. Entropy Analysis of HRV Time Series

An RR time series x=x(i),1≤i≤N, can be reconstructed by:

(1) $$u^{\left(m\right)}\left(i\right)=\left\{x\left(i\right),x\left(i+\tau \right),\ldots ,x\left(i+\left(m-1\right)\tau \right)\right\},$$

with the embedding dimension, m, and time delay parameter, τ, where  1≤i≤N−mτ.

The Chebyshev distance between two vectors in the stated space is usually used to quantify whether the two vectors are similar. The distance can be calculated by:

(2) $$d\left[u_{m}\left(i\right),u_{m}\left(j\right)\right]=max\left(\left|u_{m}\left(i\right)-u_{m}\left(j\right)\right|\right),1\leq i,j\leq N-m\tau ,$$

(1) Approximate entropy (ApEn).

If we use $N_{i}^{\left(m\right)}\left(r\right)$ to represent the number of pairs of vectors $u_{m}\left(i\right)$ and $u_{m}\left(j\right)$ that meet $d\left[u_{m}\left(i\right),u_{m}\left(j\right)\right]\leq r$, then its probability will be $C_{i}^{\left(m\right)}\left(r\right)=\frac{N_{i}^{\left(m\right)}\left(r\right)}{N-m\tau }$. The ApEn of the time series x can be calculated by [37]:

(3) $$ApEn\left(m,\tau ,r\right)=\Phi^{\left(m\right)}\left(r\right)-\Phi^{\left(m+1\right)}\left(r\right),$$

where $\Phi^{\left(m\right)}\left(r\right)=\frac{1}{N-m\tau }\Sigma_{i=1}^{N-m\tau }lnC_{i}^{\left(m\right)}\left(r\right)$ and $\Phi^{\left(m+1\right)}\left(r\right)$ is the counterpart of $\Phi^{\left(m\right)}\left(r\right)$ when the embedding dimension is equal to m+1.

(2) Sample entropy (SampEn).

Define $A_{i}^{\left(m\right)}\left(r\right)=\frac{N_{i}^{\left(m\right)}\left(r\right)}{N-m\tau -1}$ as the percentage of the number of pairs that meet $\left\{d\left[u_{m}\left(i\right),u_{m}\left(j\right)\right]<r,1\leq i,j\leq N-m\tau ,j\neq i\right\}$. The SampEn can be calculated by [20]:

(4) $$SampEn\left(m,\tau ,r\right)=-ln\frac{\Psi^{\left(m+1\right)}\left(r\right)}{\Psi^{\left(m\right)}\left(r\right)},$$

where $\Psi^{\left(m\right)}\left(r\right)=\frac{1}{N-m\tau }\Sigma_{i=1}^{N-m\tau }A_{i}^{\left(m\right)}\left(r\right)$ (i.e., the average of $A_{i}^{\left(m\right)}\left(r\right)$ over 1≤i≤N−mτ), and $\Psi^{\left(m+1\right)}\left(r\right)$ is the counterpart of $\Psi^{\left(m\right)}\left(r\right)$ when the embedding dimension is equal to m+1.

(3) Fuzzy entropy (FuzzyEn).

FuzzyEn replaces $A_{i}^{\left(m\right)}\left(r\right)$ (i.e., the percentage of the vector pairs that meet $d\left[u_{m}\left(i\right),u_{m}\left(j\right)\right]<r$) with the average degree of membership. Specifically, for a given fuzzy membership function $e^{-ln\left(2\right){\left(x/r\right)}^{2}}$, $A_{i}^{\left(m\right)}=\Sigma_{j=1,j\neq i}^{N-m\tau }e^{ln\left(2\right){\left(d∕r\right)}^{2}}$ is used [38,39]. FuzzyEn can be obtained by plugging $A_{i}^{\left(m\right)}\left(r\right)$ into the calculation of $\Psi^{\left(m\right)}\left(r\right)$ and $\Psi^{\left(m+1\right)}\left(r\right)$ in SampEn.

(4) Distribution entropy (DistEn).

The DistEn calculates the Shannon entropy of all distances to take full advantage of the inter-vector distances $\left\{d\left[u_{m}\left(i\right),u_{m}\left(j\right)\right],1\leq r,j\leq N-m\tau \right\}$. In DistEn, a histogram-based approach is used to estimate the empirical probability density function of the distance matrix (with the main diagonal elements excluded). If we denote the probability of each bin by $\left\{p_{t},t=1,2,\ldots ,B\right\}$ wherein B is the bin number in the histogram, then DistEn can be defined by the following formula [18]:

(5) $$DistEn\left(m,\tau ,B\right)=-\frac{1}{log_{2}\left(B\right)}\sum_{t=1}^{B}p_{t}log_{2}\left(p_{t}\right)$$

(5) Conditional entropy (CE).

The full range of x is coarse-grained with a fixed number of ξ values marked from 0 to ξ−1. It renders x(i) sequences of symbols $\hat{x}\left(i\right),i=1,2,\ldots ,N$. Here, ξ represents the quantization level. $u_{m}\left(i\right)$ and $u_{m}\left(j\right)$ are defined by:

(6) $$\begin{array}{c} u_{m}\left(i\right)=\left[\hat{x}\left(i\right),\hat{x}\left(i-\tau \right),\ldots \hat{x}\left(i-\left(m-1\right)\tau \right)\right]\\ u_{m+1}\left(j\right)=\left[\hat{x}\left(j\right),u_{m}\left(j-\tau \right)\right] \end{array},$$

respectively, where (m−1)τ+1≤i,j≤N. The vectors $u_{m}\left(i\right)$ and $u_{m}\left(j\right)$ can be rewritten in decimal format as:

(7) $$\begin{array}{c} {\left\{u_{m}\left(i\right)\right\}}_{10}=\hat{x}\left(i\right)\xi^{m-1}+\hat{x}\left(i-\tau \right)\xi^{m-2}+\ldots +\hat{x}\left(i-\left(m-1\right)\tau \right)\xi^{0}=w_{i}\\ {\left\{u_{m+1}\left(i\right)\right\}}_{10}=\hat{x}\left(j\right)\xi^{m}+{\left\{u_{m}\left(j-\tau \right)\right\}}_{10}=z_{i} \end{array},$$

thus rendering $u_{m}\left(i\right)$ a series of integer numbers, $w_{i}$, ranging from zero to $\left(\xi -1\right)\overset{m-1}{\underset{i=1}{\sum }}\xi^{i}$ and $u_{m}\left(j\right)$ a series of integer numbers, $z_{j}$, ranging from zero to $\left(\xi -1\right)\overset{m}{\underset{j=1}{\sum }}\xi^{j}$. CE can be computed by [38,40]:

(8) $$CE\left(m,\tau \right)=SE\left(z_{j}\right)-SE\left(w_{i}\right)+perc\left(m\right)SE\left(1\right),$$

where SE(⋅) calculates the Shannon entropy of a specific distribution; perc(m) is the percentage of $w_{i}$ pattens found only once in the data set and SE(1) means the Shannon entropy of the quantized series $\hat{x}\left(i\right)$.

(6) Permutation entropy (PermEn).

PermEn evaluates the irregularity based on a comparison of neighboring values in the state space. Each vector in the state space is re-arranged in ascending order to produce a permutation vector π. The frequency of each element $\pi_{j},1\leq j\leq m!$ is denoted as $p_{j}\left(m,\tau \right)$. Then, the PermEn can be calculated by [23,38]:

(9) $$PermEn\left(m,\tau \right)=-\frac{1}{log_{2}m!}\sum_{j=1}^{m!}p_{j}\left(m,\tau \right)log_{2}\left[p_{j}\left(m,\tau \right)\right]$$

For ApEn [20], SampEn [20], and FuzzyEn [39], the following parameters were used: *m* = 2, *r* = 0.2*σ*, wherein *σ* was the standard deviation of the time series, and *τ* = 1. The parameters were set at *m* = 2, *τ* = 1, and *B* = 64 for DistEn [41]; *m* = 2, *τ* = 1, and *ξ* = 6 for CE [40]; and *m* = 3 for PermEn [42]. The ApEn, SampEn, FuzzyEn, PermEn, and CE are direct measures of the irregularity, whereas the DistEn is sensitive to the change of complexity of time series [18]. The preprocessed RR intervals were used to calculate the six entropy measures.

### 2.3. Linear Measures of HRV Time Series

Many of the traditional methods commonly used in HRV studies are also used to investigate sleep staging. They are the mean of RR intervals (mRR), standard deviation of RR intervals (SDNN), root mean square of successive RR interval differences (RMSSD), standard deviation of successive RR interval differences (SDSD), percentage of successive RR intervals that differ by more than 50-ms (pNN50), percentage of successive RR intervals that differ by more than 30-ms (pNN30), Poincaré plot standard deviation perpendicular to the line of identity (SD1, $\;\mathrm{SD}1=\sqrt{VAR\frac{RR_{i}-RR_{i+1}}{\sqrt{2}}}$, where *VAR* is the variance), Poincaré plot standard deviation along the line of identity (SD2, $\mathrm{SD}2=\sqrt{VAR\frac{RR_{i}+RR_{i+1}}{\sqrt{2}}}$), ratio of the SD1-to-SD2 ratio ($\mathrm{SD}1/\mathrm{SD}2=\frac{SD1}{SD2}$), area of the ellipse that represents the total HRV (S=π×SD1×SD2), total power (TP), power of the very low frequency band 0.0033–0.04 Hz (VLF), power of the low-frequency band 0.04–0.15 Hz (LF), power of the high-frequency band 0.15–0.4 Hz (HF), normalized low frequency (nLF), normalized high frequency (nHF), and ratio of LF-to-HF power (LF/HF) [43]. Using the preprocessed RR intervals, the linear measures were computed.

### 2.4. Statistical Analysis

Almost everyone’s sleep data contains five sleep stages, which are W, N1, N2, N3, and REM, and a sleep stage with the same label can also correspond to multiple people. Therefore, the values of different entropies belonging to the same sleep stage were averaged within each participant. Linear mixed-effect models were performed to examine the differences in the six entropy measures across different sleep stages. The mixed model is a generalized model of linear regression by allowing random effects with respect to each subject. We treated subject as a random effect to allow a subject-specific intercept. The sleep stage was used as a fixed effect (reference level: W). The statistical significance was accepted at an alpha level of 0.05. All statistical analyses were performed using the Matlab software (Ver. R2020a, The MathWorks Inc., Natick, MA, USA) with the fitlme function.

### 2.5. Sleep Staging

The XGBoost, a classification tool commonly used in sleep studies [44,45], was selected as the classifier for sleep staging. We developed three distinct XGBoost classifiers to evaluate the performance of the six entropy measures for three different classification tasks, respectively, as shown below:

(1) a three-class classification task to differentiate among W, NREM, and REM;
(2) a four-class classification task to differentiate among W, light sleep (LS, combined N1 and N2), deep sleep (DS, or N3), and REM;
(3) a five-class classification task to differentiate among W, N1, N2, N3, and REM. Different models were trained for each classification task and then tested.

The classification accuracy (*Acc*) and Cohen’s kappa (*κ*) were used to evaluate the performance of sleep staging using the six entropy measures as well as the commonly used linear indices. *Acc* is defined as Acc=n/N, where n is the number of correct stages and N is the number of all annotations of the test set. The Cohen’s kappa (*κ*) can be computed by

(10) $$\kappa =\left(p_{o}-p_{e}\right)/\left(1-p_{e}\right)$$

where $p_{o}$ is the overall classification accuracy that is equal to *Acc*; $p_{e}=\sum_{k=1}^{q}p_{k+}p_{+k}$, where $p_{k+}$ and $p_{+k}$ indicate the percentage of data classified into the k category and the percentage of data with the true label, respectively.

A total of 23 features were used for training, including 6 entropy measures as well as the 17 commonly used linear methods. An 8-fold cross validation was performed with 14 people for training, and the remaining 2 were used for testing. During the training process, in every iteration of the eight-fold cross validation, we set aside two people from the training set for model validation and hyperparameter optimization. Model hyperparameters, i.e., the max depth, learning rate, subsample, subsample ratio of columns when constructing each tree, and regularization coefficients, were tuned using a Bayesian optimizer [46]. We used an early stopping strategy to avoid overfitting, i.e., the training would stop when the performance on validation set was not improved for 20 consecutive rounds. We also applied a class weight for controlling the balance of positive and negative weights during training to handle class imbalances. In order to explore what features contributed most and assess whether entropy-based analyses were important for each classification task, we additionally used a model explanation method called the SHapley Additive exPlanation (SHAP) value to explain the XGBoost models and to quantify the importance (i.e., the weight or contribution) of each feature on each sleep stage classification. SHAP is a game-theoretic approach that provides a theoretically justified method for the allocation of the coalition output among the members of the coalition using the classic Shapley values [47]. The exact Shapley values were computed by estimating the change in the expected prediction output of the model when specific input features were missing.

## 3. Results

### 3.1. The Results of Entropy Indices Using 270-s Epochs

**Figure 2:**
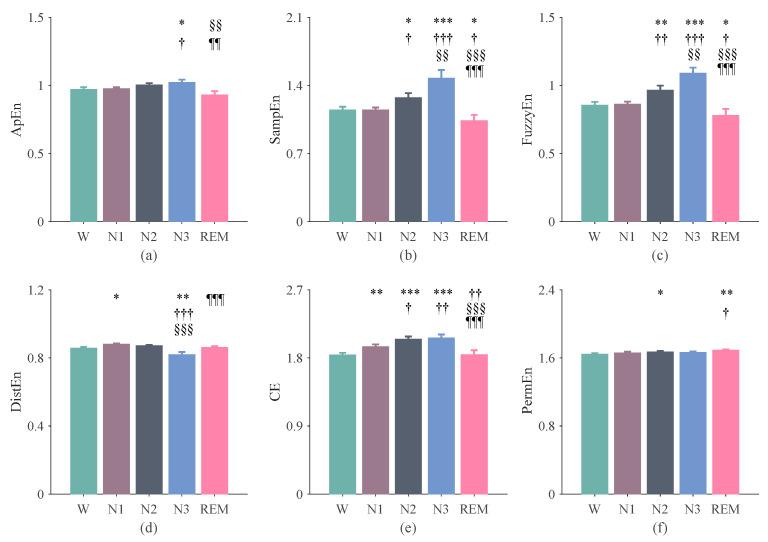
Entropy results of the RR intervals computed based on 270-s epochs. (**a**) ApEn. (**b**) SampEn. (**c**) FuzzyEn. (**d**) DistEn. (**e**) CE. (**f**) PermEn. The error bars indicate the standard error in each stage. *: W vs. N1, N2, N3, and REM; ^†^: N1 vs. N2, N3, and REM; ^§^: N2 vs. N3 and REM; ^¶^: N3 vs. REM. The number of symbols indicates the degree of significant difference, e.g., *, ^†^: *p* < 0.05; **, ^††^, ^§§^, ^¶¶^: *p* < 0.01; ***, ^†††^, ^§§§^, ^¶¶¶^: *p* < 0.0001.

The irregularity of RR interval time series showed a tendency of increasing from W to sleep and with the depth of sleep. Specifically, CE was higher in N1 than in W (Figure 2e; *p* < 0.01); SampEn, FuzzyEn, and CE were all higher in N2 than those in N1 (Figure 2b,c,e; all *p* < 0.05); SampEn and FuzzyEn were both higher in N3 than those in N2 (Figure 2b,c; both *p* < 0.001). The irregularity decreased from N3 to REM, as suggested by ApEn, SampEn, FuzzyEn, and CE (Figure 2a–c,e; all *p* < 0.01). The complexity of the RR intervals, as shown by DistEn, was the lowest during N3 (Figure 2d; compared with other stages: all *p* < 0.01). Note that PermEn did not show significant differences, except that it was higher in REM than in W or N1 (Figure 2f; *p* < 0.05).

### 3.2. The Results of Entropy Indices Using 300-s Epochs

**Figure 3:**
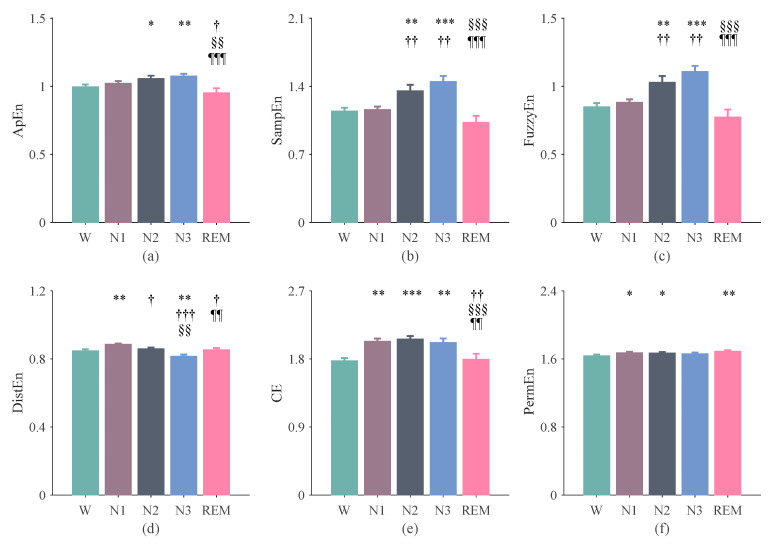
Entropy results of the RR intervals computed based on 300-s epochs. (**a**) ApEn. (**b**) SampEn. (**c**) FuzzyEn. (**d**) DistEn. (**e**) CE. (**f**) PermEn. The error bars indicate the standard error in each stage. *: W vs. N1, N2, N3, and REM; ^†^: N1 vs. N2, N3, and REM; ^§^: N2 vs. N3 and REM; ^¶^: N3 vs. REM. The number of symbols indicates the degree of significant difference, e.g., *, ^†^: *p* < 0.05; **, ^††^, ^§§^, ^¶¶^: *p* < 0.01; ***, ^†††^, ^§§§^, ^¶¶¶^: *p* < 0.0001.

Similarly, the irregularity of RR interval time series also demonstrated a tendency of increasing from W to sleep and with the depth of sleep. Specifically, CE was higher in N1 than in W (Figure 3e; *p* < 0.05); SampEn and FuzzyEn were both higher in N2 than in N1 (Figure 3b,c; *p* < 0.01). The irregularity decreased from N3 to REM, as suggested by ApEn, SampEn, FuzzyEn, and CE (Figure 3a–c,e; all *p* < 0.01). The complexity of the RR intervals, as shown by DistEn, was increased from W to N1, decreased to N2, and was lowest during N3 (Figure 3d; all *p* < 0.05). PermEn still did not demonstrate clear differences, except that it was higher in N1, N2, and REM than in W (Figure 3f; *p* < 0.05).

### 3.3. Sleep Staging

Based on epochs of 270-s, the accuracies of the models with entropy features improved by 0.9%, 3%, and 1.7% for 5 classes, 4 classes, and 3 classes, respectively, as compared with those from the models without entropy features. Based on epochs of 300-s, there were also some improvements in terms of accuracy, i.e., the accuracy increased by 0.4%, 1.5%, and 2%, respectively, for the three classification tasks. The Cohen’s kappa, *κ,* also had a certain degree of improvements, as shown in Table 1, especially for the 4- and 3-class classification tasks. The classification performances of 5 classes and 3 classes using 300-s epochs were much better than those using 270-s epochs. For the 4-class classification, the Cohen’s kappa, *κ,* of using 300-s epochs was significantly higher than that of using 270-s epochs, whereas the *Acc* did not show any significant difference between them.

**Figure 4:**
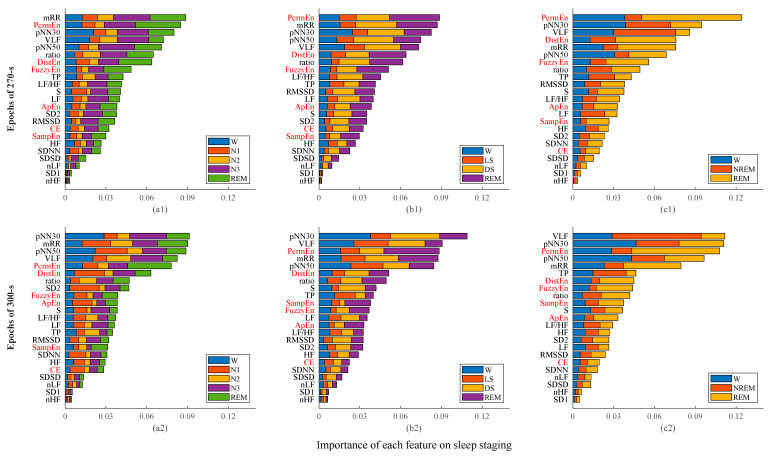
Importance of input features on sleep stage classification. Upper panels: results using 270-s epochs. Lower panels: results using 300-s epochs. Left panels (**a1**,**a2**) five-class classification. Middle panels (**b1**,**b2**) four-class classification. Right panels (**c1**,**c2**) three-class classification. The abscissa value corresponding to each feature is the weight that the feature plays in the classification.

The importance of each feature is shown in Figure 4. Based on epochs of 270-s, PermEn, DistEn, and FuzzyEn played important roles in all three classification tasks, especially in distinguishing REM and N3 from other stages. Among the six entropy measures, ApEn, SampEn, and CE had the least importance in all three classification tasks. The ApEn and SampEn performed better when using 300-s epochs, whereas PermEn, DistEn, and FuzzyEn still showed greater importance, and the CE still indicated the least importance. By using 300-s epochs, PermEn and FuzzyEn had a greater ability to differentiate REM, and DistEn was better at distinguishing N1 from other stages in the 5-class classification and at distinguishing N3 and REM from other stages for the 4- and 3-class classification.

## 4. Discussion

Entropy is a tool to quantify the complexity or irregularity of times series. It has been widely used in analyzing cardiac dynamics [18,39,48,49]. Although some studies have conducted comparisons of entropy indices of RR interval time series in certain sleep stages [24,25], it is not well-studied how they change throughout a whole night’s sleep, which may be helpful for sleep staging using ECG signals. In this paper, six entropy measures, specifically, ApEn, SampEn, FuzzyEn, DistEn, CE, and PermEn, were computed for RR interval time series based on sliding windows of 270-s or 300-s length. In addition, how the entropy measures performed in sleep staging compared to traditional linear time- and frequency-domain measures was examined using XGBoost models.

Based on the results from both 270-s and 300-s epochs, SampEn, FuzzyEn, or CE significantly increased from W to N3 and decreased in REM, whereas DistEn first increased from W to N1, decreased in N2, decreased further in N3, and, finally, increased in REM. ApEn, SampEn, FuzzyEn, CE, and PermEn showed that the irregularity of the RR intervals increased with the depth of sleep, and it decreased in the REM stage. However, DistEn demonstrated an opposite changing direction. The reason is two-fold: (1) irregularity as assessed by ApEn-based algorithms (including SampEn, FuzzyEn, and CE) is not equivalent to complexity. It is believed that complex systems are neither too random nor too regular [50,51,52] but are at a critical state of ‘complex’. The irregularity of the system increases with the degree of randomness whereas complexity does not. (2) DistEn is shown to assess the complexity of the time series based on prior simulation studies [18,50]. There has been a long debate in what exactly complexity is in the field. In general, there is not yet a single definition of complexity; the irregularity is believed to be one aspect of it. In the simulation study of DistEn, previous researchers demonstrated that its changing direction was the same to the theoretical change of the complexity of the synthetic data, and a potential explanation is that DistEn fully quantitates the distribution property of the inter-vector distances in the state space [18,53]. Therefore, although DistEn showed a different changing direction compared with the other five entropy measures, they were not contradictory to each other. In addition, there are studies to quantify complexity from another perspective, for example, multifractality [54]. Multifractality has a different theoretical basis, which may also have important implications for studying the dynamic characteristics of RR interval time series during sleep. Future studies are planned to further investigate this.

Our results are also in line with the studies of Togo et al. [55] and Bunde et al. [56], which demonstrated a perturbed fractal organization of HRV during NREM sleep. In the current study, significant differences were indicated between W and REM by SampEn, FuzzyEn (decreased), which is consistent with previous studies [26,57], and by PermEn (increased), which has also been reported before [24]. By studying the continuous transition from REM to W, a previous study has also shown that SampEn was lower in W than in REM in sleep apnea patients [58]. Therefore, it is likely that the complexity of REM just before waking is different from other REM stages. Besides, there may also be difference between tonic and phasic REMs, which is worthy of further research.

The accuracy and the Cohen’s kappa, *κ*, for the 3-class classification task with epochs of 300-s were significantly higher than those with 270-s epochs, whereas the accuracy for the 4-class classification task showed no statistical significance between them. Apparently, due to the “messy” epochs being excluded, the RR intervals with the length of 300-s almost belonged to the same sleep stage. However, this stringent exclusion criterion may result in low real-time performance in sleep staging. It is even possible that no data can be used for a fairly long period of time, especially for people with unstable sleep structures or sleep disorders. How to balance the performance and the need for real-time feedback should be an important research topic in the future.

Table 2 summarizes the performance reported in other related works. Unfortunately, the performance of our current study is the worst in all three classification tasks. One apparent reason is that different databases were used. The results in this article may be affected by the potential inclusion of people with sleep disorders, such as sleep apnea. The features and classification methods may also lead to the differences. For instance, Li et al. [31] used cardiorespiratory coupling and a convolutional neural network for sleep staging, and they achieved accuracies of 75.4% and 65.9%, respectively on two databases. Mustafa et al. [30] achieved an accuracy of 77.0% using 132 HRV features based on a long short-term memory neural network model. The third reason may be that we did not try to optimize the training and input features. By comparison, Surantha et al. [59] selected different features for every sample for staging that achieved an accuracy of 71.52%. This is, however, difficult to implement in practical applications. Ebrahimi et al. [60] did not consider sleep stage N1 in training due to the lack of available epochs. Wei et al. [61] used a 10-fold cross validation approach by epochs rather than by recordings, which had overfit issues if epochs from the same subject appeared in both training and testing sets.

It should be noted that the aim of the current study was not to improve the accuracy of sleep staging or to propose a sleep staging algorithm that works better than existing approaches. We aimed to conduct a systematic study on the complexity of HRV of different sleep stages and at the same time explore the importance of entropy measures in sleep staging. The results showed that there were significant differences in the irregularity or complexity of the RR interval in different sleep stages (Figure 2 and Figure 3). The results in Figure 4 showed that the entropy features ranked relatively high in the feature importance sequence for sleep staging.

Although the addition of the entropy measure did not significantly improve the classification effect, the results still suggested that entropy played an important role in sleep staging, especially PermEn, DistEn, and FuzzyEn, as demonstrated by the importance of these features in all three classification tasks. It could be that one or several of the time- and frequency-domain measures have a certain degree of correlation with the entropy metrics. Still, these results indicate that it is possible to obtain comparable results by using only entropy or using just the first few important indicators. These observations may help optimize the feature selection or reduce the dimension of input features for future studies. Regarding the classification with 3, 4, and 5 classes, the order of the importance of the features of 270-s and 300-s was different, and the importance of each feature to the same sleep stage was also different. This may be caused by mixed stages in the 270-s epochs but not in 300-s epochs because every feature would be affected by the information contained in the analyzed RR interval time series. Besides, the algorithm of each entropy measure is also subject to further improvement. For example, Liu et al. [64] and Ji et al. [65] improved fuzzy entropy that may better account for the global similarity of vectors and the tolerance of the degree of similarity. We will also test their performance on sleep staging in future.

Limitations of this study include: (1) The sample size was relatively small. The number of subjects is a key factor for training machine learning models. The training data further dropped when constructing 300-s epochs. For example, the data of “slp67x” only had 77 min of sleep data and had no REM stage. (2) The dataset was imbalanced. The imbalance between the numbers of epochs in NREM and wake or REM was quite noticeable. To avoid biased evaluation, the Cohen’s kappa, a measure of agreement that factors out agreement by chance due to the imbalance in the amounts of data in different sleep stages, was used to assess the performance of classification rather than using only the accuracy. A better way to handle imbalanced datasets is based on either up sampling the minor class or down sampling the major class. We will further explore this in future work. Ideally, a larger dataset is required to verify our observations, further investigate the difference in cardiac dynamics across different sleep stages, or to optimize the machine learning model for sleep staging. Achieving accurate real-time sleep staging is important for future implementation in practice. This also calls for further improvement of the entropy algorithms towards better handling the analysis of short-term data, for example, 30-s heartbeat intervals.

## 5. Conclusions

In this current study, the complexity or irregularity of RR intervals during different sleep stages was examined, and how they could help with automatic sleep staging was evaluated. The results suggested that during NREM sleep the complexity of RR intervals decreased (or the irregularity increased). Besides, the complexity increased during REM (or the irregularity decreased). The automatic sleep staging models, although they indicated only modest increases after including entropy measures, suggested greater importance than most of the traditional measures. Future work on automatic sleep staging using ECG may prioritize the feature selections based on the use of entropy measures. Besides, the current study also calls for an effort to further improve the statistical properties of the existing entropy measures in order to accommodate the analysis of ultra-short time series (such as 30-s) for better sleep staging.

## Figures and Tables

**Table 1:** Performance of the three classification tasks using XGBoost models based on 270-s and 300-s epochs using linear HRV features + entropy features and using only linear HRV features.

	Classes	*Acc* (%)		*κ* (a.u.)	
		Only Linear HRV Features	Linear HRV Features + Entropy Features	Only Linear HRV Features	Linear HRV Features + Entropy Features
270-s	5	41.2 ± 6.6	42.1 ± 7.4	0.17 ± 0.08	0.17 ± 0.10
	4	56.1 ± 9.1	59.1 ± 8.9	0.22 ± 0.15	0.25 ± 0.16
	3	59.1 ± 8.2	60.8 ± 9.5	0.23 ± 0.15	0.27 ± 0.17
300-s	5	53.9 ± 13.5 *	54.3 ± 14.3 *	0.29 ± 0.17 *	0.29 ± 0.19 *
	4	61.4 ± 12.1	63.1 ± 13.3	0.35 ± 0.20 *	0.36 ± 0.24 *
	3	65.5 ± 9.9	67.5 ± 11.6 *	0.37 ± 0.19 *	0.40 ± 0.21 *

Data are expressed as means ± standard deviations. *Acc*: accuracy; *κ*: Cohen’s kappa. *: *p* < 0.05 for the comparison between performance using 300-s epochs and 270-s epochs.

**Table 2:** Comparison between previous studies and the current study.

Work	Classes	*Acc* (%)
Yasue et al. [28]	5	66
Our work	5	54.3
Li et al. [31]	4	75.4/65.9 ^a^
Mustafa et al. [30]	4	77
Surantha et al. [59]	4	71.52
Ebrahimi et al. [60]	4	89.32
Tanida et al. [62]	4	56
Our work	4	63.1
Wei et al. [61]	3	77
Li et al. [31]	3	81.6/75.3 ^a^
Yücelbaş et al. [63]	3	76.79
Our work	3	67.5

^a^ results were achieved using two databases.

## Data Availability

The data presented in this study are openly available in Physionet: https://physionet.org/content/slpdb/1.0.0/ (accessed on 15 November 2021).
